# Genotype-Phenotype Associations in Patients With Type-1, Type-2, and Atypical *NF1* Microdeletions

**DOI:** 10.3389/fgene.2021.673025

**Published:** 2021-06-08

**Authors:** Gergely Büki, Anna Zsigmond, Márta Czakó, Renáta Szalai, Gréta Antal, Viktor Farkas, György Fekete, Dóra Nagy, Márta Széll, Marianna Tihanyi, Béla Melegh, Kinga Hadzsiev, Judit Bene

**Affiliations:** ^1^Department of Medical Genetics, Clinical Center, Medical School, University of Pécs, Pécs, Hungary; ^2^Szentágothai Research Centre, University of Pécs, Pécs, Hungary; ^3^1st Department of Pediatrics, Semmelweis University, Budapest, Hungary; ^4^2nd Department of Pediatrics, Semmelweis University, Budapest, Hungary; ^5^Department of Medical Genetics, Faculty of Medicine, University of Szeged, Szeged, Hungary; ^6^Genetic Laboratory, Szent Rafael Hospital of Zala County, Zalaegerszeg, Hungary; ^7^Full member of the European Reference Network on Genetic Tumour Risk Syndromes (ERN GENTURIS) – Project ID No. 739547, Pécs, Hungary

**Keywords:** copy number variation, type-1 *NF1* microdeletion, type-2 *NF1* microdeletion, atypical *NF1* microdeletion, 17q11.2 deletion syndrome, array-CGH, multiplex ligation-probe dependent amplification, *NF1* gene

## Abstract

Neurofibromatosis type 1 is a tumor predisposition syndrome inherited in autosomal dominant manner. Besides the intragenic loss-of-function mutations in *NF1* gene, large deletions encompassing the *NF1* gene and its flanking regions are responsible for the development of the variable clinical phenotype. These large deletions titled as *NF1* microdeletions lead to a more severe clinical phenotype than those observed in patients with intragenic *NF1* mutations. Around 5-10% of the cases harbor large deletion and four major types of *NF1* microdeletions (type 1, 2, 3 and atypical) have been identified so far. They are distinguishable in term of their size and the location of the breakpoints, by the frequency of somatic mosaicism with normal cells not harboring the deletion and by the number of the affected genes within the deleted region. In our study genotype-phenotype analyses have been performed in 17 mostly pediatric patients with *NF1* microdeletion syndrome identified by multiplex ligation-dependent probe amplification after systematic sequencing of the *NF1* gene. Confirmation and classification of the *NF1* large deletions were performed using array comparative genomic hybridization, where it was feasible. In our patient cohort 70% of the patients possess type-1 deletion, one patient harbors type-2 deletion and 23% of our cases have atypical *NF1* deletion. All the atypical deletions identified in this study proved to be novel. One patient with atypical deletion displayed mosaicism. In our study *NF1* microdeletion patients presented dysmorphic facial features, macrocephaly, large hands and feet, delayed cognitive development and/or learning difficulties, speech difficulties, overgrowth more often than patients with intragenic *NF1* mutations. Moreover, neurobehavior problems, macrocephaly and overgrowth were less frequent in atypical cases compared to type-1 deletion. Proper diagnosis is challenging in certain patients since several clinical manifestations show age-dependency. Large tumor load exhibited more frequently in this type of disorder, therefore better understanding of genotype-phenotype correlations and progress of the disease is essential for individuals suffering from neurofibromatosis to improve the quality of their life. Our study presented additional clinical data related to *NF1* microdeletion patients especially for pediatric cases and it contributes to the better understanding of this type of disorder.

## Introduction

Neurofibromatosis type 1 (NF1; MIM#162200), also known as von Recklinghausen disease, is an autosomal dominant disorder caused by loss-of-function mutations in the neurofibromin 1 (*NF1)* gene. The incidence of NF1 at birth is approximately 1 in 2500-3000 and the disease frequency shows no gender or racial predilection (Lammert et al., 2005; Uusitalo et al., 2015). The typical clinical features of NF1 are the hyperpigmented skin macules, called as café-au-lait spots (CALs), freckling of the axillary and inguinal regions, the pathognomonic neurofibromas and Lisch nodules. The neurofibromas are mostly benign tumors, localized on or under the skin (Huson and Hughes, 1994). They consist of a mixed cell types including Schwann cells, perineural cells, mast cells and fibroblasts. However, neurofibromatosis has a tremendous spectrum of clinical variability, including skeletal abnormalities, vascular disease, central nervous system tumors and cognitive dysfunction (attention deficit, learning disabilities) as well. Skeletal abnormalities such as dysplasia of the long bones are also characteristic for NF1 patients. Many features increase in frequency with aging and shows age-dependent manifestations. Moreover, strong intra- and interfamilial phenotypic variability can be observed among individuals carrying the same pathogenic mutations (Jett and Friedman, 2010).

Neurofibromin 1 gene is located on the long arm of the chromosome 17 (17q11.2) and codes for neurofibromin, a tumor suppressor that functions in the RAS/MAPK and mTOR pathways and controls the cell growth and proliferation (Jett and Friedman, 2010). The penetrance is complete and the mutation rate is high. Most of the intragenic *NF1* mutations are of paternal origin. Half of the known patients inherit the mutation, and the other half have a spontaneous mutation. Novel mutations occur primarily in paternally derived chromosomes, and the probability of these mutations increases with the paternal age (Stephens et al., 1992). A great number of germline mutations are intragenic and their effect causes a truncated neurofibromin (Park and Pivnick, 1998). Currently approximately 2000 mutations (nonsense, frameshift, point mutations etc.) are dispersed through the gene (Abramowicz and Gos, 2014).

The general NF1 population is mostly affected by point mutations or small indels, although a number of cases reported large deletions encompassing the *NF1* gene and its flanking regions. These large deletions titled as *NF1* microdeletions lead to a more severe clinical phenotype than those observed in patients with intragenic *NF1* gene mutations. These severe clinical features include large numbers of early-onset neurofibromas, cognitive deficits, dysmorphic features and an increased risk for the development of malignant peripheral nerve sheath tumors (MPNSTs) (Kehrer-Sawatzki et al., 2017).

Approximately 5-10% of NF1 patients have large deletions and the numbers are continuously increasing as a result of technological innovations (Cnossen et al., 1997; Kluwe et al., 2004; Zhang et al., 2015). Four major types of *NF1* microdeletions (type 1, 2, 3 and atypical) have been identified so far. The main difference among them are the breakpoint location, the size of the deletion, and the number of the affected genes within the deleted region (Kehrer-Sawatzki et al., 2017). The most frequent form is the type-1 *NF1* microdeletion, which is 1.4 Mb long and includes 14 protein-coding genes and four microRNA genes as well (Dorschner et al., 2000; Lopez-Correa et al., 2001). Type-1 deletions account for 70-80% of all large *NF1* deletions (Pasmant et al., 2010; Messiaen et al., 2011). Type-2 *NF1* deletions are less common than type-1 and they represent ca. 10-20% of all large *NF1* deletions (Mautner et al., 2010; Pasmant et al., 2010; Messiaen et al., 2011). Type-2 deletions are 1.2 Mb in size and result in the deletion of 13 genes. In contrast to type-1 and type-2 *NF1* deletions, type-3 *NF1* deletions are very rare, their occurrence is around 1-4% of patients with *NF1* microdeletions (Bengesser et al., 2010; Pasmant et al., 2010; Messiaen et al., 2011). This type of deletion spans 1 Mb and leads to the loss of 9 protein coding genes.

Type-1, 2, and 3 NF1 microdeletions are generated by non-allelic homologous recombination (NAHR) between low-copy repeats (LCRs) during either meiosis (type-1, type-3), or mitosis (type-2) (Dorschner et al., 2000; Jenne et al., 2001; Lopez-Correa et al., 2001; Bengesser et al., 2010; Pasmant et al., 2010; Roehl et al., 2010; Zickler et al., 2012; Hillmer et al., 2016). Type-1 cases are usually maternally inherited germline deletions (Neuhausler et al., 2018), while type-2 ones are predominantly of postzygotic origin (Kehrer-Sawatzki et al., 2004; Steinmann et al., 2008; Vogt et al., 2012). Besides these three types of recurrent microdeletions, atypical *NF1* deletions have been identified in a number of patients. In atypical deletions non-recurrent breakpoints have been discovered, thereby the size of the deletion and the number of the affected genes also vary (Kehrer-Sawatzki et al., 2003, 2005, 2008; Mantripragada et al., 2006; Pasmant et al., 2010; Messiaen et al., 2011). Non-homologous end joining mechanism has been associated mostly with atypical deletions (Venturin et al., 2004a). However, either aberrant DNA double strand break repair and/or replication, and retrotransposon-mediated mechanisms have also been supposed to be involved in the background of their formation (Vogt et al., 2014). Atypical microdeletions may occur approximately in 8-10% of all patients with NF1 microdeletions (Pasmant et al., 2010).

Somatic mosaicism with normal cells not harboring large *NF1* deletion can be observed with different frequencies in different types of *NF1* deletions. This phenomenon is rare among type-1 deletions, vast majority (more than 95%) of the patients with type-1 deletion is non-mosaic (Messiaen et al., 2011; Summerer et al., 2019). Contrast to type-1 deletion, somatic mosaicism is quite common in type-2 *NF1* deletions, it occurs in at least 63% of all type-2 deletions (Vogt et al., 2012). Atypical *NF1* deletions also display mosaicism frequently. In a study reported by Vogt et al. (2014), approximately 60% of the cases were associated with somatic mosaicism (Vogt et al., 2014). It is worth to note that somatic mosaicism with normal cells without the deletion has a considerable effect on the disease phenotype, however it is difficult to assess its presence.

In addition to the extent of somatic mosaicism, the age of the patients is also an important confounding factor in phenotypic comparisons of NF1 patient cohort, since many symptoms are progressive in onset and some of them appears later in life (Cnossen et al., 1998).

Several research groups have investigated different aspects of *NF1* microdeletions, however only a few studies presented profound clinical examinations. Here we report clinical and genotype data from 17 patients, mainly (82%) children and adolescents, carrying different types of microdeletion. One of the patients with atypical deletion showed somatic mosaicism. The aim of our study was to characterize the detected deletions in our patient cohort and elucidate genotype-phenotype correlations through clinical data collection.

## Materials and Methods

### Participants

Between 2009 and 2019, our laboratory tested 640 unrelated patients with suspected neurofibromatosis. After Sanger sequencing of the *NF1* gene or NGS analyses of *NF1, NF2, KIT, PTPN11, RAF1, SMARCB1, SPRED1* genes no disease-causing mutations have been identified in 252 patients. Of these, 17 patients (7 females, 10 males; mean age at time of examination:12.9 years, age range:2-36 years) with large *NF1* deletion were identified by MLPA and were enrolled into this study. Our patient cohort mostly (14 out of 17) consisted of children between the ages of 2 and 17. Two patients inherited the deletion from their mothers (patients 85 and 260), while in the remaining 15 patients the deletions had *de novo* origin based on the negative MLPA results of the parents or the absence of a clinically affected parent. However, in the latter case low grade or tissue specific mosaicism cannot be ruled out. The mother of patient 260 (patient 134) was clinically affected as well, therefore she was also included in the analysis. The mother of patient 85 was sine morbo. As a control, age and sex matched 33 patients (14 females, 19 males; mean age at the time of examination: 15.2 years, age range:6 months-47 years) with intragenic *NF1* mutations were enrolled into the study as well.

The study was approved by the ethics committee of the University of Pecs (Protocol 8581-7/2017/EUIG). Written informed consent was obtained from all patients or their legal guardians and peripheral blood samples were collected. All experiments were performed in accordance with the Helsinki Declaration of 1975 and with the Hungarian legal requirements of genetic examination, research and biobanking.

All of the patients fulfilled the diagnostic NIH criteria for NF1. Main clinical characteristics of our patient cohort are summarized in Table 1. Phenotypic data was obtained from our Genetic counseling unit and from our collaborator clinicians.

**TABLE 1:** Clinical features of our patients with different type of *NF1* microdeletions.

	**Deletion type**	**Type 1**								**Type 1**				**Type 2**	**Atypical**			
	**Applied method**	**aCGH**								**MLPA**				**aCGH**	**aCGH**	**MLPA**		
	**Patients**	**68/NF**	**115/NF**	**255NF**	**428NF**	**467/2016**	**532/NF**	**629/NF**	**761/NF**	**9/NF**	**271/NF**	**387/NF**	**483/NF**	**85/NF**	**556/NF**	**125/NF**	**134/NF**	**260/NF**
	**Gender**	**M**	**F**	**M**	**M**	**F**	**M**	**F**	**M**	**M**	**M**	**F**	**M**	**F**	**M**	**F**	**F**	**M**
	**Age of onset**	**26 y**	**5 mo**	**at birth**	**at birth**	**N/A**	**12 y**	**at birth**	**at birth**	**at birth**	**at birth**	**at birth**	**5 y**	**1 mo**	**6.5 y**	**at birth**	**3 y**	**at birth**
	**Age at examination**	**36 y**	**9 y**	**14 y**	**5 y**	**9 y**	**14 y**	**4.5y**	**9 y**	**21 y**	**4 y**	**17 y**	**7.5 y**	**13 y**	**10 y**	**2 y**	**40 y**	**8 y**
Dysmorphic features	Facial dysmorphism	X	X	X	X	-	X	X	-	X	-	-	X	-	-	-	-	X
	Hypertelorism	X	X	X	X	-	X	X	-	-	-	-	X	-	-	-	X	X
	Facial asymmetry	-	-	-	-	-	X	-	X	X	-	-	-	-	-	-	-	-
	Coarse face	X	-	X	X	-	X	X	X	X	-	-	X	X	-	-	-	-
	Broad neck	-	-	X	-	-	-	-	-	-	-	-	-	-	-	-	-	-
	Large hands, feet	-	X	X	X	-	X	X	X	X	-	-	X	X	-	-	-	-
Skin manifestations	CALs	X	X	X	X	X	X	X	X	X	X	X	X	X	X	X	X	X
	Freckling	-	X	X	X	X	-	X	X	X	X	X	X	-	X	X	-	X
	Excess soft tissue	-	-	X	X	-	-	X	-	X	-	-	-	X	-	-	-	-
	SBC neurofibromas	X	X	X	X	-	-	-	X	-	X	X	-	-	-	-	X	-
	CT neurofibromas	-	-	-	-	-	-	-	-	X	-	-	-	-	-	-	-	-
	PL neurofibromas*	-	-	-	-	-	-	-	-	X	-	X	-	-	-	-	-	-
Education and behavior problems	SDiCD	X	-	X	X	X	X	X	X	X	-	-	X	-	-	-	-	X
	Learning difficulties	X	-	X	-	X	X	X	X	X	-	X	X	X	-	-	-	-
	Speech difficulties	-	-	X	X	X	X	X	X	-	-	X	X	-	-	-	-	-
	IQ < 70	-	-	-	–	-	-	-	-	X	-	-	-	-	-	-	-	-
	ADHD	-	-	-	X	-	-	-	-	X	-	-	-	-	-	-	-	-
Skeletal manifestations	Skeletal anomalies	X	X	X	X	X	X	X	X	X	X	X	-	X	X	X	X	X
	Scoliosis	X	-	X	-	-	X	-	-	X	X	-	-	X	-	-	X	-
	Pectus excavatum	-	X	-	X	-	X	-	-	X	X	-	-	-	-	X	-	X
	Bone cysts	X	n.d.	-	n.d.	n.d.	-	-	-	-	-	-	-	-	-	-	-	-
	Joint hyperflexibility	-	-	-	X	-	-	-	-	-	-	-	-	-	-	-	-	-
	Macrocephaly	-	X	X	X	X	-	X	X	-	-	X	-	X	-	-	-	X
Neurological manifestations	Muscular hypotonia	X	-	X	-	-	-	-	X	-	-	-	-	-	-	-	-	-
	Headache	-	-	-	X	-	-	-	-	-	-	-	-	-	-	-	-	-
	Coordination problem	-	-	X	X	X	-	-	X	-	-	-	-	-	-	-	-	-
	MPNST	X	-	-	-	-	-	-	-	-	-	X	-	-	-	-	-	-
	Spinal neurofibromas	-	n.d.	n.d.	n.d.	-	-	n.d.	X	-	-	X	-	n.d.	-	-	n.d.	n.d.
	T2 hyperintensities	X	X	X	X	-	-	X	X	X	X	X	X	X	-	X	n.d.	X
Ocular manifestations	Visual disturbance	-	-	-	-	-	X	-	-	-	-	X	-	X	-	-	-	-
	Lisch nodules	-	-	X	-	-	-	-	-	X	-	X	-	X	-	-	-	-
	Strabismus	-	-	-	-	-	X	-	-	-	-	-	X	-	-	-	-	-
	OPG	-	-	-	-	-	-	X	-	-	-	X	-	-	X	-	-	X
Development. problems	Tall stature	-	X	X	-	-	X	X	X	X	-	X	-	-	-	-	-	-

CALs, café-au-lait spots; CT/SBC/PL, cutaneous/subcutaneous/plexiform neurofibroma; SDiCD, significant delay in cognitive development; ADHD, Attention deficit hyperactivity disorder; MPNST, malignant peripheral nerve sheath tumors; OPG, Optic Pathway Gliomas. * means externally observable plexiform neurofibroma. -, absent; X, present, n.d., not determined.

### Sample Preparation and MLPA Analysis

DNA was isolated from peripheral blood leukocytes with E.Z.N.A.^®^ Blood DNA Maxi kit (Omega BIO-TEK, Norcross, United States). The concentration and purity of extracted DNAs were measured with the NanoDrop 2000 spectrophotometer (Thermo Fisher Scientific, Waltham, MA, United States).

Multiplex ligation-dependent probe amplification (MLPA) assays were performed for screening large deletions or duplications in *NF1* gene using the commercially available SALSA MLPA kits P081-D1 and P082-C2 (MRC-Holland, Amsterdam, The Netherlands). The two probemixes contained together one probe for each exon, three probes for exon 1, one probe for intron 1, and two probes for the exons 15, 21, 23, 51, and 58 of the *NF1* gene. Additionally, one upstream and one downstream probe of *NF1* gene and two probes for the *OMG* gene (located within intron 36 of *NF1* gene) were applied. Moreover, SALSA MLPA kit P122-D1 *NF1* area mix was used for the examination of the contiguous genes in the flanking regions. The probemix contained 20 probes for 16 genes (*MYO1D*, *PSMD11*, *ZNF207*, *LRRC37B*, *SUZ12*, *UTP6*, *RNF135*, *ADAP2*, *ATAD5*, *CRLF3*, *SUZ12P*, *CPD*, *BLMH*, *TRAF4*, *PMP22*, *ASPA*), which were localized upstream and downstream as well. Besides, it also contained probes for five distinct *NF1* exons (1, 17, 30, 49, 57). According to the manufacturer’s instructions, a total of 100–200 ng of genomic DNA of each patient and the same amount of three control genomic DNA was used for hybridization. Amplification products were separated by capillary electrophoresis on an ABI 3130 Genetic Analyzer (Life Technologies, United States) and the results were analyzed using Coffalyser software (MRC-Holland, Amsterdam, Netherlands). Each MLPA signal was normalized and compared to the corresponding peak area obtained from the three control samples. Deletions and duplications of the targeted regions were suspected when the signal ratio exceeded 30% deviation. Positive results were confirmed by repeated MLPA experiments and further investigated with array CGH.

### Whole Genome Array Comparative Genomic Hybridization Analysis

Array comparative genomic hybridization (aCGH) was performed using the Affymetrix CytoScan 750 K Array. Genomic DNA samples were digested, ligated, amplified, fragmented, labeled, and hybridized to the CytoScan 750 K Array platform according to the manufacturer’s instructions. The raw data were analyzed by ChAS v2.0 Software (Affymetrix, Thermo Fisher Scientific, Waltham, MA).

### CNV Interpretation

DNA sequence information of the identified CNVs refer to the public UCSC database (GRCh37/hg19). CNV interpretation was performed with the help of the following databases and websites: DECIPHER (Database of Chromosomal Imbalance and Phenotype in Humans using Ensembl Resources) (Firth et al., 2009), DGV (Database of Genomic Variants), Ensembl and ECARUCA (European Cytogeneticists Association Register of Unbalanced Chromosome Aberrations) (Vulto-van Silfhout et al., 2013). The estimated size of the deletions and the estimated breakpoints were assessed using the known locations of the last proximal and first distal deleted probes.

### Somatic Mosaicism Determination

In patients examined by aCGH assay, allele difference plot and B allele frequency (BAF) plot were evaluated together with Log2 ratios and weighted Log2 ratios with the help of ChAS software to assess the presence and extent or absence of somatic mosaicism. In those samples investigated by MLPA, the ratio values for each MLPA probe were used to assess mosaicism. Values between 0.4-0.6 were considered as non-mosaic deletion, values around 0.7 or up to 0.8 were considered as mosaic deletion.

### Clinical Investigation

Phenotypic features of the 17 microdeletion and the 33 control patients were collected using the same standardized questionnaire collection protocol in four HCPs (health care provider). The same patient was always examined and followed up by the same clinician. Most features were identified by physical examination. Dysmorphic features were assessed by expert clinical syndromologist following international guidelines^1^ (Allanson et al., 2009; Hall et al., 2009). Lisch nodules and other ocular manifestations were diagnosed by an ophthalmologist. To evaluate childhood overgrowth age and race-related percentile curve was applied. All the patients were investigated by cranial MRI. To evaluate intellectual functions, developmental delay and learning disabilities, patients were assessed by various psychological tests appropriate to their age (Walter Strassmeier’s developmental scale: ages between 0 and 5 years (Strassmeier, 1980), Bayley Scales test (BSID-III): ages between 1 and 42 months (Bayley, 2006), Budapest Binet test: ages between 3 and 14 years (Bass et al., 1989)). When IQ was not measured, it was estimated to be > 70 based on the fact that the patient attended a regular kindergarten or school (with special educational needs). ADHD was diagnosed following international guidelines^2^. The term “speech difficulties” was used in those cases when the patient did not speak or he or she had a problem with the language content, language structure and expressive vocabulary and grammar. We assigned it to delayed language development and not neurological symptoms (dysarthria or orofacial dyskinesis).

### Statistical Analysis

All statistical analyses were performed with SPSS version 27 (SPSS Inc,. Chicago, IL, United States). Two-tailed Fisher’s exact test was used to assess whether there is a difference in the frequency of clinical features between patients with type-1 *NF1* microdeletion and patients with intragenic *NF1* mutations. A difference with *p* < 0.05 was considered as significant.

## Results

### Characterization of the *NF1* Microdeletions

**FIGURE 1:**
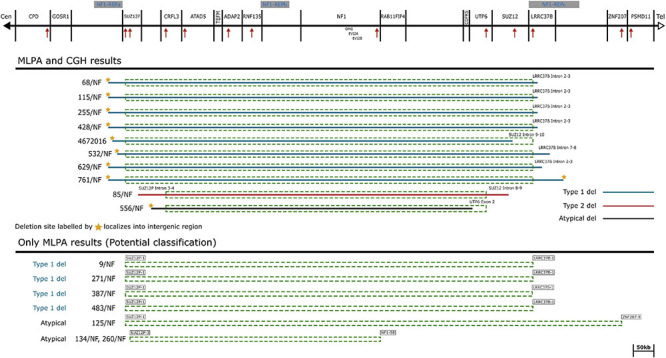
Schematic representation of the *NF1* gene and flanking regions. The affected genes and NF1-REP regions are schematically displayed at the top of the figure. Localization of MLPA probes are demonstrated by red arrows. Solid lines symbolize the deletion range with known breakpoints determined by aCGH probes. Dotted rectangles correspond to the deleted range determined by MLPA probes. Deletion types are marked by colored solid lines, blue: type-1 deletions, red: type-2 deletion and black: the suggested atypical deletion. The last probes contained by the deletion are explicitly displayed at the ends of the deletion ranges.

**FIGURE 2:**
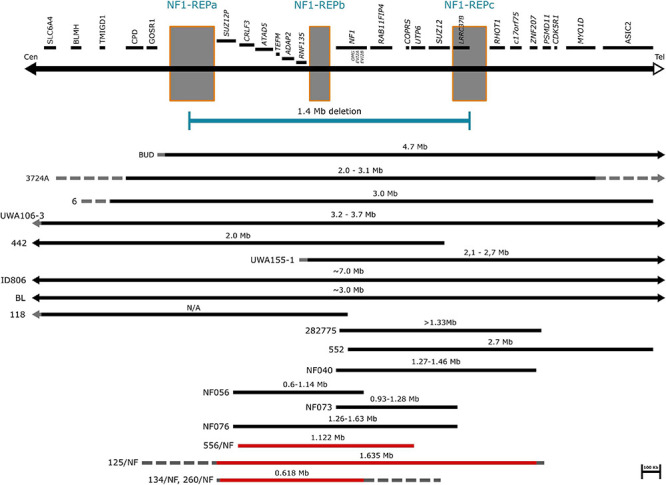
Schematic representation of atypical NF1 deletions. The affected genes and NF1-REP regions schematically are displayed at the top of the figure. Horizontal black bars represent the already known atypical *NF1* cases. Solid lines indicate the deleted regions, dotted lines indicate the possibly deleted regions. Horizontal red bars refer to our cases. Solid lines represent the deleted regions, while dotted lines suggest the potential deletion range.

A total of 252 patients in whom mutation analysis did not find any pathogenic *NF1* point mutations or intragenic insertions/deletions were screened for large *NF1* rearrangements by MLPA. Heterozygous deletions of the entire *NF1* gene and its flanking regions were identified in 17 patients using SALSA P081/082 assay. To determine the contiguous genes involved in the deletion, the SALSA P122 assay was applied. As a result, majority of our cases (12/17) had type-1 deletion. Moreover, the MLPA analysis revealed atypical deletions in 5 patients. The estimated proximal and distal breakpoints, preceding and following marker locations and the estimated size of the deletions identified by MLPA are summarized in Supplementary Table 1. To confirm the MLPA results, array comparative genomic hybridization (aCGH) analyses were performed in 10 patients (8 patients with type-1 and 2 patients with atypical deletions). The estimated location of proximal and distal breakpoints, preceding and following markers and the estimated size of the deletions determined by aCGH are summarized in Supplementary Table 2. The classification by MLPA and by aCGH were found to be the same in eight cases (7 type-1 deletion and 1 atypical). In patient 85/NF the aCGH finally revealed the existence of type-2 deletion although the MLPA showed atypical deletion. In patient 4672016 the aCGH test showed atypical deletion whereas MLPA detected a type-1 deletion, finally we considered this patient has type-1 deletion. The discrepancy between the MLPA and aCGH results in these cases may originate from the different localization of the probes. Type-2 deletions are characterized by breakpoints located within *SUZ12* gene and its pseudogene *SUZ12P*. SALSA P122 probe set contains only one probe for *SUZ12* gene (SUZ12-10: localized within exon 10) and 2 probes for *SUZ12P* pseudogene (SUZ12P-3, SUZ12P-1: probe localization within exon 3 and exon 1, respectively). The breakpoints of the deletion detected in our patient (85/NF) were localized within the region covered by *SUZ12* and *SUZ12P* probes of P-122 set. The applied CytoScan 750K chip contains more probes, at least 50 and 7 for *SUZ12* and *SUZ12P*, respectively. Therefore, aCGH was capable to identify this type-2 deletion. Breakpoints of type-1 deletions are located within the low-copy repeats NF1-REPa and NF1-REPc. In patient 4672016 the estimated proximal breakpoint detected by aCGH can be found within NF1-REPa and the estimated distal breakpoint detected by MLPA can be found within NF1-REPc, therefore we considered 4672016 patient as having type-1 deletion. In the remaining 7 cases (4 patients with type-1 and three with atypical deletions), aCGH tests were not feasible due to the quality of the available samples. After all, 8 type-1 deletions, 4 potential type-1 deletions (altogether 12 type-1 deletions), one type-2 deletion and 3 atypical deletions in four patients were identified in our patient cohort. No type-3 microdeletion was found in our patients. Among the type-1 deletions aCGH analyses revealed identical estimated breakpoints in four cases with an approximately 1.37 Mb deletion size. Among atypical cases three distinct novel deletions were detected. Patient 134/NF and 260/NF are close relatives (mother and child), so they possess the same deletion. The results of our MLPA and aCGH analyses with the localization of the MLPA probes are visualized in Figure 1. Novel atypical deletions identified in this study, together with the already known atypical NF1 cases, are demonstrated in Figure 2 and Tables 4, 5. Two out of three novel atypical deletions were identified by MLPA. SALSA P122 probe set contains 23 probes within the 17q region and the distance between the adjacent probes are quite variable from 11 kb up to 1500 kb. The preceding markers of the estimated proximal breakpoint and the following markers of the estimated distal breakpoint are localized far from the breakpoint boundaries. The distance between the preceding markers and the estimated proximal breakpoints are ca. 270 kb and 27 kb in case 125/NF and 260/NF (134/NF), respectively. The distance between the following markers and the estimated distal breakpoints are ca. 80 kb and 500 kb in case 125/NF and 260/NF (134/NF), respectively. MLPA is able to identify only estimated location of breakpoints, the exact localization of the breakpoints can be determined precisely by breakpoint-spanning PCR (Summerer et al., 2018). In our cases the actual breakpoints are presumably located somewhere between two MLPA probes. Therefore, the regions in proximal direction from the first probe or in distal direction from the last probe affected by the deletion until the adjacent probe are suggested as potential deleted region and represented in Figure 2 with dotted lines.

### Assessment of Somatic Mosaicism

Among 10 patients investigated by aCGH, only one subject (556/NF) with atypical *NF1* microdeletion displayed somatic mosaicism with an extent of ca. 30%. In 7 patients examined by MLPA, the ratio values do not imply the presence of any mosaicism. However, neither aCGH, nor MLPA measurements are capable to detect low-grade mosaicism below 20% due to the nature of these techniques. In this study we investigated only blood samples, so to completely rule out mosaicism, examination of additional tissues such fibroblast, buccal or urine cells are necessary. In type-1 *NF1* microdeletion the occurrence of somatic mosaicism is known to be very rare (Summerer et al., 2019), so based on our results our type-1 patients can be considered as non-mosaic cases. The only one patient with type-2 deletion inherited the deletion from her mother, consequently she does not possess somatic mosaicism. Anyway, this is compatible with the aCGH result as well. Among our four patients with atypical *NF1* deletion, the results indicated ca. 30% mosaicism in only one case (556/NF). Patient 260/NF inherited the deletion from his mother, therefore this patient is considered as non-mosaic. His mother (134/NF) is supposed to be a non-mosaic case as well, since she has a positive family history (her mother and her grandmother were also affected, however, without laboratory diagnosis) and the MLPA results (peak ratios were between 0.49-0.55) also supported this assumption. MLPA peak ratios were between 0.49 and 0.55 also for patient 125/NF, therefore we supposed this patient to be a non-mosaic as well.

### Clinical Characterization of Our Patients With Different Type of *NF1* Microdeletion

Several clinical features and neuropsychological manifestations belonging to eight major categories were selected for consideration for genotype-phenotype association analysis (Table 1). The frequency of each clinical feature that appeared in patients with type-1 *NF1* microdeletion is compared with frequencies observed in our control group, i.e., patients with intragenic *NF1* mutation (Supplementary Tables 4, 5).

### Dysmorphic Features

Facial dysmorphism was described in 9 of the 17 patients investigated (53%). It was present in 8 out of 12 patients with type-1 *NF1* deletion (67%) and in 1 out of 4 atypical *NF1* deletion (25%) patient cohort. The prevalence of hypertelorism was similar to that of facial dysmorphism, however the distribution among the deletion types was different. This clinical feature was found to roughly the same extent in type-1 deletion and atypical deletion cases (58% vs 50%, respectively). Facial asymmetry was noted only in 3 out of 12 patients with type-1 deletion. Coarse facial appearance was frequent in type-1 deletion patients (8 out of 12 patients, 67%), it was present also in the type-2 deletion patient, though it was absent in our atypical cases. Large hand and feet seem to be a characteristic dysmorphic feature of *NF1* microdeletion patients as well, since the majority of our patients with type-1 deletion (67%, 8 out of 12) showed this trait and it was also noted in the type-2 patient. Dysmorphic features were rare events in our intragenic *NF1* patient population. Of the examined dysmorphic traits only hypertelorism and facial asymmetry were found with the frequency of 18% (6 out of 33 controls) or 6% (2 out of 33 controls), respectively.

### Skin Manifestations

Café-au-lait spots (CALs) were observed in each patient in our study regardless of the type of the deletion they have. Axillary and inguinal freckling occurred also in high frequency in our patient cohort. It was more common within the type-1 deletion group, 10 out of 12 patients (83%) presented this skin manifestation. In atypical deletion group 3 out of 4 patients (75%) displayed this feature, however, it was absent in the type-2 deletion patient. Moreover, another skin manifestation, i.e. excess soft tissue in hands and feet was observed among our patients, though at a lower frequency. In type-1 deletion group it was noted in 4 out of 12 patients (33%), it developed in a patient with type-2 deletion also, in contrast, it was not found in the atypical deletion patients. Skin manifestations are characteristic for intragenic *NF1* patients as well. CALs were presented in 91% (30 out of 33) of our patients and the frequency of axillary and inguinal freckling was 52% (17 out of 33 controls).

### Neurofibromas and Other Tumors

Subcutaneous neurofibromas were found more common in type-1 deletion patient cohort compared to type-2 and atypical groups. They were observed in 7 out of 12 patients (58%) with type-1 deletion, in 1 out of 4 patients (25%) with atypical microdeletion, though none occurred in the patient with type-2 deletion. The prevalence of cutaneous neurofibromas appears to be less frequent in our patient cohort, it was observed in only one patient with type-1 deletion. However, it is important to mention that 14 out of 17 patients were children and furthermore 10 out of 14 were under 10 years old at the age of examination.

Externally observable plexiform neurofibromas were seen in only 2 patients with type-1 deletion, in a 21-year-old boy and a 17-year-old girl. None of the patients with type-2 or atypical microdeletions presented this type of neurofibromas. However, this is worth to mention that whole-body MRI was not performed routinely in our patients, therefore we have no information about the internally occurring plexiform neurofibromas.

Spinal neurofibromas were found in the type-1 microdeletion group only, however, within this group, the prevalence was low, it developed in 2 out of 12 patients (17%). However, the observed low occurrence is probably the result of the fact, that spinal MRI is not part of the routine procedure in our patient management.

Optic pathway glioma (OPG) was detected by MRI in 4 patients and it was not symptomatic in any of these cases. It was more common in the atypical group with 50% prevalence. Moreover, it developed in 2 out of 12 patients (17%) with type-1 deletion but it was absent in the patient with type-2 deletion. Among the control patients 2 symptomatic and 2 asymptomatic OPG were observed.

Malignant peripheral nerve sheath tumors (MPNST) were observed in 2 of our patients, both belonging to type-1 deletion group. None of the patients with type-2 or atypical microdeletions displayed this type of tumor. MPNSTs show age-related penetrance and our patient cohort consisted of mainly children under 17 years, therefore it is not surprising to detect low occurrence among our patients. However, both patients presenting MPNSTs were adult or nearly adult (36 years and 17 years old, respectively), consequently the frequency of this type of tumor was high (50%, 2 out of 4) among adult patients.

Among our intragenic *NF1* patients, subcutaneous fibromas were found with 30% (10 out of 33) frequency, the occurrence of cutaneous and plexiform neurofibromas were 18% (6 out of 33) or 6% (2 out of 33), respectively. Spinal neurofibromas were observed in 3% (1 out of 33) of our patients. Moreover, 12% (4 out of 33) of this patient cohort developed optic pathway glioma, however, no malignant peripheral nerve sheath tumors occurred.

### Skeletal Anomalies

Anomalies of the skeletal system were detected in almost all of our patients (94%, 16 out of 17). The most frequent skeletal anomaly was macrocephaly, which was observed in 9 out of 17 patients (53%). This clinical feature was common in type-1 microdeletion cohort with 58% prevalence, whereas in atypical cohort only one patient (25%) presented this symptom.

Scoliosis was noted in 7 out of 17 patients studied here (41%). It was more frequent in patients with type-1 *NF1* microdeletion than in patients with other type of *NF1* microdeletions. Interestingly, there were only 2 patients who presented scoliosis together with macrocephaly.

Pectus excavatum was observed in 35% of our patient cohort. In contrast to scoliosis, this skeletal anomaly was more frequently observed in patients with atypical microdeletion (50%) as compared to type-1 deletion group (33%).

Bone cysts were found in only one patient with type-1 microdeletion.

None of our patient displayed pes cavus, however, other foot deformities such as pes planus was observed in 3 patients.

Interestingly, skeletal anomalies were the leading manifestations in our patient with type-2 deletion. She had macrocephaly, scoliosis, bilateral dislocation of the elbow and wrist joint. Moreover, absorption of the tibial malleolus was observed and she developed osseous malignancy as well.

Skeletal anomalies were less frequently observed in the intragenic *NF1* patient group (33%). Of these, scoliosis occurred most frequently with 21% prevalence. Macrocephaly and pectus excavatum were noted in 9% of the patients and 3% of them presented pes cavus.

### Ocular Manifestations

Ocular manifestations were observed in 7 of 17 our patients (41%). Lisch nodule, one of the characteristic hallmarks of type 1 neurofibromatosis, was noted only in 3 out of 12 patients with type-1 deletion and in the patient with type-2 deletion, however, it was not observed in the atypical patient cohort. Moreover, other ocular manifestations, such as visual disturbance, strabismus and proptosis were noticed in 2 patients with type-1 deletion and in the type-2 deletion patient. One of the patients had hypermetropia, while the others had myopia. The frequency of ocular manifestations was similar in the intragenic *NF1* patient cohort. Lisch nodule was noted in 21% (7 out of 33) of the patients and 15% (5 out of 33) presented visual disturbances as well. One patient had myopia, two patients had hypermetropia, and two other patients had anisometropia. However, strabismus was not observed.

### Neuropsychological Manifestations

Significant delay in cognitive development and general learning difficulties were observed with high frequency (75%, 9 out of 12) in type-1 patients. Furthermore, along with the previous features, speech difficulties occurred in 67% (8 out of 12) of this patient group. One patient had an IQ below 70 and 2 patients showed attention deficit hyperactivity disorder (ADHD). IQ measurement was performed in only two among our type-1 patients (761/NF IQ:77, 9/NF IQ:47), however, all of our pediatric patients attended regular kindergarten or school, except the one with IQ = 47, and five of them have special educational needs. Therefore, we supposed these patients are not intellectually disabled, so we marked them as negative for IQ < 70 criteria in Table 1. Majority of these neuropsychological features were not found in atypical patient cohort (patient 556/NF IQ:89) and in the type-2 patient. Only a significant delay in cognitive development was noted in 25% (1 out of 4) of atypical patients and the type-2 patient suffered from general learning difficulties.

Structural brain abnormalities were not observed in our patients, however, T2 hyperintensities were found in the majority of our patients. It was present with 75% (9 out of 12) prevalence in type-1 deletion patient cohort, with 25% (1 out of 4) prevalence in atypical group and also in the patient with type-2 deletion. Nevertheless, we did not find any correlation between the age of our patients and the T2 signal intensities.

Muscular hypotonia and coordination problems (25% and 33%, respectively) were documented in patients with type-1 deletion. None of these neurological symptoms were found in our type-2 and atypical deletion groups.

Epilepsy and nerve pain were not noted in our patients. One patient with type-1 deletion complained of headache.

Neuropsychological manifestations were not common among the patients with *NF1* intragenic mutation. 3% (1 out of 33) of our patients presented significant delay in cognitive development, speech difficulties and epilepsy. Moreover, general learning difficulties were noted with a bit higher frequency (15%, 5 out of 33). Muscular hypotonia was observed in 12% (4 out of 33) of our patients and T2 hyperintensities were found in 39% (13 out of 33) of them.

### Connective Tissue Anomalies and Cardiac Abnormalities

Connective tissue anomalies and heart abnormalities were a very rare event in our patient cohort. Hyperflexibility of joints was observed in 2 out of 12 type-1 deletion patients (17%). Such manifestation was not present in our patients with type-2 or atypical deletions. Among the cardiac abnormalities atrial septal defect was observed in one patient with atypical microdeletion. Moreover, hypertrophic cardiomyopathy was observed in one patient (8%) and patent ductus arteriosus (PDA) occurred in another patient (8%) with type-1 microdeletion. No congenital heart defect, pulmonary stenosis, ventricular septal defect, aortic stenosis, aortic dissection, mitral valve prolapses, mitral valve insufficiency, aortic valve insufficiency was found in any of the deletion groups. It should mention that two of our patients were not investigated by cardiac ultrasound.

These manifestations were rare in our patients with *NF1* intragenic mutation as well. Among the cardiac abnormalities only ventricular septal defect was observed at birth in one patient and 6% (2 out of 33) of our patients developed joint laxity.

### Other Features

Some rare clinical manifestations were observed in our patient group. Obesity, hearing impairment, immune deficiency and milk protein allergy, however it is hard to tell whether these symptoms are associated with the observed large deletion or the results of an independent event.

## Discussion

The *NF1* gene was discovered in Viskochil et al. (1990), somewhat later the first case with large *NF1* microdeletion was published in Kayes et al. (1992). Several attempts were made to establish genotype-phenotype correlations which finally suggested a more severe clinical phenotype among patients with *NF1* microdeletion than patients with intragenic *NF1* mutations. However, certain variability of clinical symptoms has been observed among individuals with *NF1* microdeletions.

In this study, we have identified 17 patients with large *NF1* microdeletion. Among them 8 proved to be a type-1 microdeletion carrier by aCGH, 4 more patients are supposed to belong to type-1 group based on MLPA results, 1 patient has type-2 deletion and 4 patients possess atypical deletions. Somatic mosaicism with an extent of ca. 30% was detected in one patient with atypical *NF1* microdeletion. Comparison of clinical characterization of our patients with the published data on intragenic and microdeletion NF1 patients was performed to reveal distinct phenotype-genotype correlations. Moreover, the frequencies of phenotypic features in our patients with *NF1* microdeletion and with type-1 deletion were compared to frequencies observed in our patients with intragenic *NF1* mutation as well (Supplementary Tables 3–5).

A similar difference was found between our patients with intragenic *NF1* mutation and *NF1* microdeletion in several clinical features when comparing to those previously published by others (Table 2). Mainly the occurrence of dysmorphic features, subcutaneous neurofibromas, skeletal anomalies and neurobehavior problems showed significant difference. Moreover, remarkable differences in certain clinical features were observed between our patients with *NF1* microdeletion and the previously published cases with large *NF1* deletions. However, it is important to emphasize that the majority of our patients (13 out of 17) were less than 15 years old at the time of the examination. There are only few studies (Kehrer-Sawatzki et al., 2020) that demonstrated pediatric clinical data, the majority of phenotypic data published previously originated mainly from adult patient populations.

**TABLE 2:** Clinical features of patients with type-1 *NF1* microdeletion.

		**Frequency in patients with**					**Frequency in *NF1***	
		**type-1 *NF1* microdeletions (%)**					**non-deleted patients (%)**	
**System involvement/manifestations**	**Clinical features**	**This study (*n* = 12)**	**Kehrer-Sawatzki et al., 2017 (*n* = 29)**	**Pasmant et al., 2010 (*n* = 44)**	**Zhang et al., 2015 (*n* = 7)**	**Bianchessi et al., 2015 (*n* = 11)**	**This study (*n* = 33)**	**Kehrer-Sawatzki et al., 2017 (*n* = 29)**
Dysmorphic features	Facial dysmorphism	67	90	54.8	43	n.d.	0	n.d.
	Hypertelorism	58	86	n.d.	n.d.	n.d.	18	n.d.
	Facial asymmetry	25	28	n.d.	n.d.	n.d.	6	8
	Coarse face	67	59	n.d.	n.d.	n.d.	0	n.d.
	Broad neck	8	31	n.d.	n.d.	n.d.	0	n.d.
	Large hands and feet	67	46	n.d.	n.d.	n.d.	0	n.d.
Skin manifestations	Café-au-lait spots	100	93	20.8	100	100	91	86-99
	Axillary and inguinal freckling	83	86	86.4	57	72.7	52	86-89
	Excess soft tissue in hands and feet	33	50	n.d.	n.d.	n.d.	0	n.d.
	Subcutaneous neurofibromas	58	76	37.2-41.8	29	45.5^#^	30	48
	Cutaneous neurofibromas	8	86	15.4-48.7	57	45.5^#^	18	38-84
	Plexiform neurofibromas	17	76	0.6	29	27.3	6	15-54
Education and behavior problems	SDiCD	75	48	n.d.	14	36.4	3	17
	General learning difficulties	75	45	85.7	n.d.	18.2	15	31-47
	Speech difficulties	67	48	n.d.	29	0	3	20-55
	IQ < 70	8	38	n.d.	14	36.4	0	7-8
	ADHD	17	33	n.d.	n.d.	0	6	38-49
Skeletal manifestations	Skeletal anomalies	92	76	31+	14	45.5+	33	31
	Scoliosis	42	43	31	0	9.1	21	10-28
	Pectus excavatum	33	31	n.d.	n.d.	n.d.	9	12-50
	Bone cysts	8	50	n.d.	n.d.	0	0	1
	Hyperflexibility of joints	8	72	n.d.	n.d.	n.d.	6	n.d.
	Pes cavus	n.d.	17	n.d.	n.d.	n.d.	3	n.d.
	Macrocephaly	58	39	11.5	14	45.5	9	24-45
Neurological manifestations	Muscular hypotonia	25	45	n.d.	n.d.	n.d.	12	27
	Epilepsy	0	7	n.d.	n.d.	0	3	4-13
	MPNST	17	21	7.1	0	*	0	2-7
	Spinal neurofibromas	17	64	n.d.	n.d.	n.d.	3	24-30
	T2 hyperintensities	75	45	n.d.	29	n.d.	39	34-79
Ocular manifestations	Visual disturbance	17	n.d.	n.d.	14	n.d.	15	n.d.
	Lisch nodules	25	93	40	14	45.5	21	63-93
	Strabismus	17	NA	n.d.	14	n.d.	0	NA
	Optic pathway gliomas	17	19	15	n.d.	0	12	11-19
Developmental problem	Tall-for-age stature	58	46	22.2	n.d.	n.d.	0	n.d.
Heart problems	Congenital heart defects	0	29	n.d.	n.d.	n.d.	0	2

n.d., not determined; NA, not assessed or no data available; ^#^no straightforward information (only referenced as neurofibroma); *it is not clear from the manuscript (it was mentioned that 18.2% of patient had tumors); + it may be higher (there were data for scoliosis and macrocephaly only); SDiCD, significant delay in cognitive development; MPNST, malignant peripheral nerve sheath tumors; ADHD, attention deficit hyperactivity disorder.

Type-1 deletion represents the largest group of *NF1* microdeletion cohort with an estimated 70-80% prevalence (Pasmant et al., 2010; Messiaen et al., 2011). The occurrence of this type of deletion among our patients was somewhat similar (70%). Significant number of articles were published on this type of deletion, however, these reports indicate that the clinical phenotype associated with *NF1* microdeletions show a certain degree of variability in the frequency of some clinical features (Table 2) (Mensink et al., 2006; Mautner et al., 2010; Pasmant et al., 2010; Bianchessi et al., 2015; Zhang et al., 2015). Dysmorphic features are common in individuals with large *NF1* deletions, whereas they occur rarely among intragenic NF1 patient population. Among these features facial dysmorphism is one of the most characteristic hallmarks of patients with *NF1* microdeletion. In our type-1 patient cohort 67% of the affected individuals possess this manifestation. At the same time in a large study performed by Mautner et al. involving 29 patients (Mautner et al., 2010), the majority of the cases (ca 90%) had facial dysmorphism. However, Pasmant and Zhang observed this feature with lower frequency (Pasmant et al., 2010; Zhang et al., 2015). Nevertheless, all of these data indicate that facial dysmorphic features are very frequent in type-1 deletions. Another dysmorphic feature which can be seen more often in microdeletion patients is the observed large hands and feet. It occurred with 67% prevalence in our patient cohort, it was observed in 46% of patients by Mautner (Mautner et al., 2010), however, it was not stated by others. Another observable difference can be seen in the number of the detected neurofibromas. Previous studies established an early-onset of neurofibromas among *NF1* microdeletion patients. While the frequency of the detected subcutaneous neurofibromas in our patients was close to that observed by others (58 vs 76%), the occurrence of cutaneous or plexiform neurofibromas was remarkably lower in our patients compared to other patient groups (8 vs. 86% and 17 vs. 76%, respectively). However, it is worth to highlight, that our patient cohort mainly consisted of children and adolescents, and 9 out of 17 were less than 10 years old at the time of examination. Cutaneous neurofibromas show age-related penetrance and they usually appear in adulthood, therefore this may contribute to the difference in frequency observed by us and by others. Nevertheless, a high frequency (60%) of cutaneous neurofibromas was observed among children by Kehrer-Sawatzki in a recent study (Kehrer-Sawatzki et al., 2020). The high prevalence of subcutaneous neurofibromas in type-1 *NF1* patients is important to consider, since they are associated with mortality in NF1 disease (Tucker et al., 2005). Patients with subcutaneous neurofibromas possess a higher risk for the development of MPNSTs. In addition, the presence of plexiform neurofibromas possess a risk for development of malignant tumor as well (Waggoner et al., 2000). More pronounced alteration can be seen in the cognitive ability. Although, significant delay in cognitive development was found more frequently in our type-1 patients, the prevalence of intellectual disability was less pronounced. Moreover, overgrowth, which is characteristic for type-1 *NF1* microdeletion, was observed as much as by others, however, connective tissue anomalies were fairly less frequent among our patients. It was common among Mautner’s patients (72%), but it was rare (8%) in our patient cohort.

Type-1 deletion harbors 14 protein coding genes and 4 microRNA genes. Some of the genes co-deleted with *NF1* may have an influence on the clinical manifestation observed in patients with *NF1* microdeletion, thus affecting the severity of the disease (Kehrer-Sawatzki et al., 2017). Haploinsufficiency of certain genes may contribute to dysmorphic facial features, overgrowth and reduced cognitive capability (*RNF135*) (Tastet et al., 2015) or heart defects (*ADAP2*) (Venturin et al., 2014), whereas others might have tumor suppressive function, thus their deletion promote tumor development (*SUZ12, ATAD5*) (Bell et al., 2011; Zhang et al., 2014). Although the size of the deletion and the gene content is almost the same in all patients with type-1 deletion, they demonstrate a notable clinical variability. This observation may suggest that differences in the unique genomic architecture of the patients may also contribute to the observed variability of the clinical phenotypes.

Type-2 deletions account for 10-20% of *NF1* large deletion cases according to previous studies. In our patient cohort one patient and her asymptomatic mother carries this type of large *NF1* deletion. Because of the missing phenotypic signs, we suppose that the mother should be a mosaic patient. In type-2 deletions existence of somatic mosaicism is a frequently observed phenomenon, these deletions arise during post-zygotic cell division and are associated with a milder clinical phenotype. Vogt et al. reported 18 patients with type-2 deletion, 16 of whom proved to be mosaic cases (Vogt et al., 2011). In another study the same research group identified 27 of 40 patients with mosaicism determined by FISH. That paper did not contain clinical information, because it was focused on the possible molecular mechanism behind type-2 deletion formation (Vogt et al., 2012). Only a few non-mosaic type-2 cases with detailed phenotype have been published so far (Table 3; Vogt et al., 2011; Zhang et al., 2015). These patients share common features, half of which can be found in our patient as well. However, some characteristic hallmarks of *NF1* microdeletion symptoms are missing from our patient’s phenotype or they are presented in a mild form. This may originate from her young age (13 years). She does not have any type of externally observable neurofibromas, cardiac manifestations, those that may manifest as early as childhood, and neurobehavioral problems, whereas these features were noted in the majority of the published cases. Moreover, frequent skin manifestation such as freckling was not observed in our patient. These traits occurred in other known type-2 patients. The unique feature of our patient is that the whole clinical picture is dominated by skeletal anomalies. She underwent a number of operations affecting the skeletal system. Moreover, absorption of the tibial malleolus was observed and she developed osseous malignancy as well. After all her clinical picture possesses many features frequently observed in patients with large *NF1* deletion. Although type-2 deletions are typically 1.2 Mb in size, the exact localization of the breakpoints are presumably different in our patient and in the published cases. This may result in the removal of certain regulatory factors which may finally lead to the observed variability in the phenotype.

**TABLE 3:** Clinical features of patients with type-2 *NF1* microdeletions.

**Clinical features of patients with type-1**			**Presence or absence of the features in patients with “non-mosaic”**			
***NF1* microdeletions (frequency observed,%)**			**type-2 *NF1* deletions**			
Patients	*n* = 29	*n* = 12	078	P. 2429	P. 2358	85/NF
Reference	Kehrer-Sawatzki et al., 2017	This study	Zhang et al., 2015	Roehl et al., 2010; Vogt et al., 2012	Roehl et al., 2010; Vogt et al., 2012	This study
CALs	93%	100%	+	+	+	+
Freckling	86%	83%	−	+	+	−
Lisch nodule	93%	25%	?	+	+	+
Cutaneous neurofibromas	86%	8%	+	+ (multiple)	−	−
Subcutaneous neurofibromas	76%	58%	+	+ (multiple)	+	−
Plexiform neurofibromas	76%	17%	−	+ (multiple)	+	−
Facial dysmorphism	90%	67%	−	+	+	−
Large hands and feet	46%	67%	N/A	+	+	+
Macrocephaly	39%	58%	−	+	+	+
Tall stature	46%	58%	N/A	−	−	−
Learning disabilities	48%	75%	?	+	+ (mild)	+
Attention deficits	33%	17%	?	+	+	−
Scoliosis	43%	42%	+	−	N/A	+
Hyperflexibility of the joints	72%	8%	N/A	+	+	−
MPNST	21%	17%	−	+	−	−
T2 hyperintensities	45%	75%	N/A	−	+	+
Muscular hypotonia	45%	25%	N/A	N/A	+	−
Congenital heart defects	21%	0%	N/A	+	+	−

*−, absent; +, present; N/A, not assessed or no data available; ? unclear result from the original article. CALs, café-au-lait spots; MPNST, malignant peripheral nerve sheath tumors.*

Atypical deletions form a heterogeneous group of *NF1* microdeletions regarding the clinical manifestations they cause as well as the size and location of the deletion. Moreover, somatic mosaicism can be frequently observed among these patients which may lead to a milder phenotype. The occurrence of atypical cases is around 8-10% among patients with *NF1* microdeletion, however, in our patient cohort we observed a higher frequency (23%) and only one patient displayed mosaicism. Around 20 patients with atypical deletion were published so far without recurrent breakpoints (Kayes et al., 1992; Upadhyaya et al., 1996; Cnossen et al., 1997; Dorschner et al., 2000; Riva et al., 2000; Kehrer-Sawatzki et al., 2003, 2005, 2008; Venturin et al., 2004a, b; Mantripragada et al., 2006; Zhang et al., 2015). In our study three distinct, novel deletions were identified. The deletions in the published cases show remarkable overlaps with those observed in our patients, though in our cases the deletions are typically smaller (Figure 2). However, the clinical pictures of the known cases show hardly any overlapping symptoms apart from the major diagnostic criteria for NF1 (Table 4). Remarkable difference can be seen in dysmorphic features, neuropsychological manifestations and the presence of various neurofibromas. Dysmorphic features such as facial dysmorphia, coarse face, facial asymmetry and large hands and feet are characteristic hallmarks of *NF1* microdeletions. They were observed in the majority of patients with type-1 *NF1* microdeletion (Table 2) and it was noted at least in half of the atypical cases identified so far, however, in our patient cohort only one patient displayed facial dysmorphia and another had hypertelorism. Moreover, these features were not observed in patients described by Zhang et al. (2015). In addition, notable divergence can be observed in the occurrence of various neurofibromas among the atypical *NF1* microdeletion patients. All the patients in Zhang’s study manifested cutaneous or plexiform neurofibromas, 6 out of 11 other published cases had various type of neurofibromas, whereas in our study only one patient has developed subcutaneous neurofibromas. This discrepancy may be related to the age of the patients. It is a known phenomenon that the number of the neurofibromas may increase with the age of the patient. Among atypical cases the majority of the patients who presented any type of neurofibromas were teenagers or young adults. In our patient cohort, which consisted of mainly children under 10 years, the only one who had subcutaneous neurofibroma was 40 years old. In addition, observable difference can be found among the neuropsychological manifestation. These features were almost absent in our patients, only one showed significant delay in cognitive development, however, moderate to severe intellectual disability or severe learning disability were noted in almost all patients carrying larger deletion than our patients. In an atypical deletion the gene content of the deleted region has an effect on the phenotypic manifestations, particularly the genes with intolerance of haploinsufficiency are likely to have pathological consequences. Table 5 summarized the haploinsufficiency intolerant genes in all cases published so far including this study. Although in 3 out of 4 patients of ours only MLPA measurements were feasible, the deletion of one more haploinsufficiency intolerant gene, namely *RAB11FIP4*, may be expected beyond those demonstrated in Table 5. The exact role of this gene in the disease pathogenesis is not clear. Previous studies (Descheemaeker et al., 2004; Ottenhoff et al., 2020) revealed that *NF1* microdeletion genotype is associated with a lower cognitive ability compared with intragenic *NF1* genotype. Co-deletion of genes adjacent to *NF1*, such as *OMG* and *RNF135* are supposed to contribute to the observed decreased cognitive ability (Kehrer-Sawatzki et al., 2017). *OMG* gene encodes the oligodendrocyte myelin glycoprotein which plays an important role in early brain development (Martin et al., 2009). Moreover, *OMG* is associated with intellectual disability and neuropsychiatric disorders (Bernardinelli et al., 2014). In addition, a rare allele of *RNF135* gene has been found with higher frequency in patients with autism (Tastet et al., 2015). Although the deletion identified in our patients encompass *OMG* and *RNF135* genes as well, our patients hardly displayed neuropsychiatric symptoms. This observation implies that beyond the *OMG* and *RNF135* deletion further factors are also necessary for the development of intellectual disability or neuropsychiatric manifestations in patients with *NF1* microdeletions. Contrary to our cases, high load of internal tumors were observed in a number of patients with larger atypical deletion. Several genes (*ATAD5, COPRS, UTP6* and *SUZ12*) in the 17q11.2 region were supposed to be involved in tumorigenesis (Kehrer-Sawatzki et al., 2017). *ATAD5* was affected in our two patients, co-deletion of *ATAD5, COPRS* and *UTP6* was observed in another one. However, none of these patients of ours developed internal tumors. Co-deletion of *ATAD5, COPRS, UTP6* and *SUZ12* genes with *NF1* may possess an increased risk for high tumor load which might lead to the observed high number of tumors in patients with larger atypical deletion. In one of our patients the atypical deletion harbors all of these four genes, however, perhaps due to her young age (i.e., 2 years) no tumors were found at the age of her examination.

**TABLE 4:** Clinical features of patients with atypical *NF1* microdeletions.

**Patient**	**Age (y)**	**Gender**	**Skin manifestations**	**Neurofibromas**	**Dysmorphic features**	**Skeletal manifestations**	**Ocular Manifestations**	**Neuropsychological manifestations**	**Other**	**References**
BUD	14; 18	N/A	CALs, F	Many CNF, SNF	Coarse face	SCS, genu valgum, joint laxity	N/A	SDiCD, ID, T2 hyperintensities	Many ST	Kehrer-Sawatzki et al., 2003
3724A	13	Female	CALs, F	Few CNF	Coarse face, FA, hypertelorism, ptosis, broad lips and nose	PE	LiN	Moderate ID	-	Cnossen et al., 1997
6	N/A	N/A	N/A	N/A	N/A	N/A	N/A	N/A	N/A	Venturin et al., 2004a, b
UWA106-3	18	Male	CALs, F	Many CNF, PNF, spinal NF	Coarse face, large hands	MA	N/A	SDiCD, IQ 46	Many ST	Dorschner et al., 2000; Kayes et al., 1992
442	18; 26	Male	CALs, F	Multiple SCNF, and many CNF, PNF	Coarse face	SCS	LiN	IQ 76, severe LD	Many ST	Kehrer-Sawatzki et al., 2005
BL	13,5	Male	CALs, F	-	FD, hypertelorism	Skeletal anomalies	-	Severe ID	-	Riva et al., 2000
ID806	3 mo; 3; 4	Male	CALs, F	-	Narrow palpebral fissures, ptosis, low set, rotated ears, prominent maxilla	-	-	Marked developmental delay, SP, seizure	-	Upadhyaya et al., 1996
UWA155-1	27	N/A	-	Multiple CNF, spinal NF	Coarse face, ptosis, large hands and feet	MA	-	Moderate ID	MPNST	Dorschner et al., 2000
118	5	Male	CALs, F	N/A	-	-	OPG	Seizure, no LD	-	Venturin et al., 2004b
282775	n.d.	N/A	CALs	-	Noonan-like FD	-	-	PD, SP	-	Mantripragada et al., 2006
552	20	Female	CALs, F	2 PNF, 4 SIN NF	Large hands and feet	PE, lumbar lordosis, pedes valgoplanus	LiN, visual disturbance	Mild ID, severe LD, SP, hypotonia	-	Kehrer-Sawatzki et al., 2008
NF040	1	Female	CALs	PNF	-	-	*	*	-	Zhang et al., 2015
NF056	60	Female	CALs, F	CNF	-	-	*	*	-	
NF073	25	Female	CALs, F	CNF	-	-	*	*	-	
NF076	36	Female	CALs	CNF	-	-	*	*	-	
556/NF	10	Male	CALs, F	-	-	Bilateral PP	OPG	-	-	this study
125/NF	2	Female	CALs, F	-	-	PE	-	-	-	
134/NF	40	Female	CALs	SCNF	Hypertelorism	SCS	-	-	-	
260/NF	8	Male	CALs, F	-	FD, hypertelorism	PE, MA	OPG	SDiCD, T2 hyperintensities	ASD	

CALs, café-au-lait spots; F, freckling; FA, facial asymmetry; FD, facial dysmorphy; CNF, cutaneous neurofibroma; SCNF, subcutaneous neurofibroma; PNF, plexiform neurofibroma; SIN NF, small intramuscular nodular neurofibroma; ST, spinal tumors; MPNST, malignant peripheral nerve sheath tumors; SDiCD, significant delay in cognitive development; ID, intellectual disability; LD, learning difficulties; SP, speech delay; PD, psychomotor delay; SCS, scoliosis; PE, pectus excavatum; MA, macrocephaly; PP, pes planus; LiN, Lisch nodule; ASD, atrial septal defect. * unclear results in the original article. NA, no data available.

**TABLE 5:** Size of the deletions and haploinsufficient genes located within the atypical *NF1* deletions.

**Patient**	**Deletion size (Mb)**	**Haploinsufficient genes (by gnomAD pLI)**	**References**
BUD	4.7	*ATAD5, NF1, OMG, RAB11FIP4, SUZ12, PSMD11, CDK5R1, ASIC2*	Kehrer-Sawatzki et al., 2003
3724A	2.0-3.1	*ATAD5, NF1, OMG, RAB11FIP4, SUZ12, PSMD11, CDK5R1, ASIC2*	Cnossen et al., 1997
6	3	*ATAD5, NF1, OMG, RAB11FIP4, SUZ12, PSMD11, CDK5R1, ASIC2*	Venturin et al., 2004a, b
UWA106-3	3.2-3.7	*ATAD5, NF1, OMG, RAB11FIP4, SUZ12, PSMD11, CDK5R1, ASIC2*	Dorschner et al., 2000; Kayes et al., 1992; Kayes et al., 1994
442	2	*ATAD5, NF1, OMG, RAB11FIP4, SUZ12*	Kehrer-Sawatzki et al., 2005
BL	∼3	*ATAD5, NF1, OMG, RAB11FIP4, SUZ12, PSMD11, CDK5R1, ASIC2*	Riva et al., 2000
ID806	∼7	*ATAD5, NF1, OMG, RAB11FIP4, SUZ12, PSMD11, CDK5R1, ASIC2*	Upadhyaya et al., 1996
UWA155-1	2.1-2.7	*NF1, OMG, RAB11FIP4, SUZ12, PSMD11, CDK5R1, ASIC2*	Upadhyaya et al., 1996
118	N/A	*ATAD5, NF1*	Venturin et al., 2004b
282775	> 1.33	*NF1, OMG, RAB11FIP4, SUZ12*	Mantripragada et al., 2006
552	2.7	*NF1, OMG, RAB11FIP4, SUZ12, PSMD11, CDK5R1, ASIC2*	Kehrer-Sawatzki et al., 2008
40	1.27-1.46*	*NF1, OMG, RAB11FIP4, SUZ12*,	Zhang et al., 2015
56	0.60-1.14*	*ATAD5, NF1, OMG*	
73	0.93-1.28*	*NF1, OMG, RAB11FIP4, SUZ12*	
76	1.26-1.63*	*ATAD5, NF1, OMG, RAB11FIP4, SUZ12*	
556/NF	1.122	*ATAD5, NF1, OMG, RAB11FIP4, SUZ12*	This study
125/NF	1.635*	*ATAD5, NF1, OMG, RAB11FIP4, SUZ12*	
134/NF	0.618*	*ATAD5, NF1, OMG*	
260/NF	0.618*	*ATAD5, NF1, OMG*	

*Results originated from MLPA probes location. The probability of loss of function (pLI) metric were provided by the gnomAD browser (https://gnomad.broadinstitute.org/). According to official description, a transcript’s intolerance to variation is measured by predicting the number of variants expected to be seen in the gnomAD dataset and comparing those expectations to the observed amount of variation. The range scales from 0 to 1, where the closer the pLI value is to 1, the more intolerant the gene appears to be to loss of function (LoF) variants. We determined as haploinsufficient a gene if the pLI value was above 0.9, which indicates extreme intolerance to LoF variants (Karczewski et al., 2020).

Genotype-phenotype analyses among our patients revealed that ones with *NF1* microdeletion more often presented dysmorphic facial features, macrocephaly, large hands and feet, delayed cognitive development and/or learning difficulties, speech difficulties, overgrowth and subcutaneous neurofibromas compared to those with intragenic *NF1* mutations. These features seemed to be characteristic for the patient group with type-1 *NF1* microdeletion, however, some of the above-mentioned traits were absent from the type-2 and atypical *NF1* microdeletion patient cohort. Our patient with non-mosaic type-2 *NF1* large deletion had only a few of the typical clinical signs: macrocephaly, large hands and feet as well as learning difficulties. On the other hand, she has a strong skeletal involvement. In our atypical *NF1* microdeletion patient cohort only the facial dysmorphism, delayed cognitive development, macrocephaly and the presence of subcutaneous neurofibromas were noted. Certain clinical symptoms such as congenital heart defects, joint laxity, muscular hypotonia and bone cysts were reported by others in type-1 *NF1* microdeletion patients (Mautner et al., 2010; Kehrer-Sawatzki et al., 2017), but these were not pronounced in our patients. It is worth to mention that manifestations of several symptoms are age dependent, therefore a comprehensive study on the clinical course of patients with different type of *NF1* microdeletion could help to establish diagnostic milestones in these patients’ group.

## Conclusion

In conclusion, in our patient cohort three different types of *NF1* microdeletion have been identified. Although these deletions were associated with different clinical manifestations, possibly due to the deleted gene contents or the deletion of other regulatory DNA elements, patients with *NF1* large deletion showed more severe clinical phenotype compared to individuals with intragenic *NF1* mutations. The identification and in some cases the classification of the *NF1* microdeletions have been feasible using MLPA, a simple, cost-effective technique. This method enabled us to recognize *NF1* microdeletion patients easily among the general NF1 patients. Our study presented additional clinical data related to *NF1* microdeletion patients especially for pediatric patients and it contributes to the better understanding of this type of disorder.

## Data Availability Statement

The original contributions presented in the study are included in the article/Supplementary Material, further inquiries can be directed to the corresponding author/s.

## Ethics Statement

The studies involving human participants were reviewed and approved by Ethics Committee of the University of Pecs (Protocol 8581-7/2017/EUIG). Written informed consent to participate in this study was provided by the participants’ legal guardian/next of kin. Written informed consent was obtained from the minor(s)’ legal guardian/next of kin for the publication of any potentially identifiable images or data included in this article.

## Author Contributions

JB conceived and designed the research. GB, RSz, GA, and MCz performed the genetic investigations. KH, AZs, MSz, GyF, VF, MT, and DN performed the patient examinations. GB and JB completed data analysis and drafted the manuscript. GB prepared the figures and tables. GB, BM, KH, and JB edited and revised the manuscript. All authors read and approved the final manuscript.

## Conflict of Interest

The authors declare that the research was conducted in the absence of any commercial or financial relationships that could be construed as a potential conflict of interest.

## Footnotes

1 http://elementsofmorphology.nih.gov/

2 https://www.nhs.uk/conditions/attention-deficit-hyperactivity-disorder-adhd/symptoms/
