# Supplementary material for: Genotype-Phenotype Associations in Patients With Type-1, Type-2, and Atypical NF1 Microdeletions
Source: Front Genet. 2021 Jun 8;12:673025. doi: 10.3389/fgene.2021.673025 (PMC8217751; doi:10.3389/fgene.2021.673025)
Supplement: Supplementary file 1 [file Table_1.docx]

**Supplementary Table 1** Estimated sizes of the deletions and genomic localizations of MLPA probes found at and near the deletion boundaries

|  | | |  | **MLPA probe positions** | | | |
| --- | --- | --- | --- | --- | --- | --- | --- |
|  |  |  | **Estimated deletion size (bp)** | **Estimated preceding marker location** | **Estimated proximal breakpoint** | **Estimated distal breakpoint** | **Estimated following marker location** |
| aCGH results acquired | Type 1 | 68/NF | 1,290,184 | CPD-ex21 (28,789,435) | SUZ12P-ex1 (29,058,406) | LRRC37B-ex1 (30,348,590) | ZNF207-ex9 (30,693,769) |
|  |  | 115/NF | 1,290,184 | CPD-ex21 (28,789,435) | SUZ12P-ex1 (29,058,406) | LRRC37B-ex1 (30,348,590) | ZNF207-ex9 (30,693,769) |
|  |  | 255/NF | 1,290,184 | CPD-ex21 (28,789,435) | SUZ12P-ex1 (29,058,406) | LRRC37B-ex1 (30,348,590) | ZNF207-ex9 (30,693,769) |
|  |  | 428/NF | 1,290,184 | CPD-ex21 (28,789,435) | SUZ12P-ex1 (29,058,406) | LRRC37B-ex1 (30,348,590) | ZNF207-ex9 (30,693,769) |
|  |  | 4672016 | 1,290,184 | CPD-ex21 (28,789,435) | SUZ12P-ex1 (29,058,406) | LRRC37B-ex1 (30,348,590) | ZNF207-ex9 (30,693,769) |
|  |  | 532/NF | 1,290,184 | CPD-ex21 (28,789,435) | SUZ12P-ex1 (29,058,406) | LRRC37B-ex1 (30,348,590) | ZNF207-ex9 (30,693,769) |
|  |  | 629/NF | 1,290,184 | CPD-ex21 (28,789,435) | SUZ12P-ex1 (29,058,406) | LRRC37B-ex1 (30,348,590) | ZNF207-ex9 (30,693,769) |
|  |  | 761/NF | 1,290,184 | CPD-ex21 (28,789,435) | SUZ12P-ex1 (29,058,406) | LRRC37B-ex1 (30,348,590) | ZNF207-ex9 (30,693,769) |
|  | Type 2 | 85/NF | 1,077,985 | SUZ12P-ex3 (29,085,164) | CRFL3-ex3 (29,124,383) | UTP6-ex14 (30,202,368) | SUZ12-ex10 (30,315,425) |
|  | Atypical | 556/NF | 1,077,985 | SUZ12P-ex3 (29,085,164) | CRFL3-ex3 (29,124,383) | UTP6-ex14 (30,202,368) | SUZ12-ex10 (30,315,425) |
|  | | |  | | | | |
| No aCGH results could be acquired | Type 1 | 9/NF | 1,290,184 | CPD-ex21 (28,789,435) | SUZ12P-ex1 (29,058,406) | LRRC37B-ex1 (30,348,590) | ZNF207-ex9 (30,693,769) |
|  |  | 271/NF | 1,290,184 | CPD-ex21 (28,789,435) | SUZ12P-ex1 (29,058,406) | LRRC37B-ex1 (30,348,590) | ZNF207-ex9 (30,693,769) |
|  |  | 387/NF | 1,290,184 | CPD-ex21 (28,789,435) | SUZ12P-ex1 (29,058,406) | LRRC37B-ex1 (30,348,590) | ZNF207-ex9 (30,693,769) |
|  |  | 483/NF | 1,290,184 | CPD-ex21 (28,789,435) | SUZ12P-ex1 (29,058,406) | LRRC37B-ex1 (30,348,590) | ZNF207-ex9 (30,693,769) |
|  | Atypical | 125/NF | 1,635,363 | CPD-ex21 (28,789,435) | SUZ12P-ex1 (29,058,406) | ZNF207-ex9 (30,693,769) | PSMD11-ex2 (30,773,999) |
|  |  | 134/NF | 617,963 | SUZ12P-ex1 (29,058,406) | SUZ12P-ex3 (29,085,164) | NF1 exon 58 (29,703,127)* | UTP6-ex14 (30,202,368) |
|  |  | 260/NF | 617,963 | SUZ12P-ex1 (29,058,406) | SUZ12P-ex3 (29,085,164) | NF1 exon 58 (29,703,127)* | UTP6-ex14 (30,202,368) |

Genome coordinates shown in table 1 are extracted from the Product Description SALSA®MLPA® Probemix P122-D1 NF1 AREA. The positions were localized on human genome assembly hg18 (NCBI build 36) which were converted by NCBI Genome Remapping Service and Lift Genome Annotations to genome assembly hg19 (GRCh37). The median position of the probe sequences provided by the description were used to demonstrate exact genomic coordinates.

*Genomic positions were extracted from Product Description SALSA®MLPA® Probemix P081-D1 NF1 mix 1 & P082-C2 NF1 mix 2.
