# Supplementary material for: Genotype-Phenotype Associations in Patients With Type-1, Type-2, and Atypical NF1 Microdeletions
Source: Front Genet. 2021 Jun 8;12:673025. doi: 10.3389/fgene.2021.673025 (PMC8217751; doi:10.3389/fgene.2021.673025)
Supplement: Supplementary file 2 [file Table_2.docx]

**Supplementary Table 2** Estimated sizes of the deletions and genomic localizations of aCGH probes found at and near the deletion boundaries

|  |  | **aCGH probe positions** | | | | |
| --- | --- | --- | --- | --- | --- | --- |
|  |  | **Estimated deletion size (bp)** | **Preceding marker location** | **Proximal breakpoint** | **Distal breakpoint** | **Following marker location** |
| **Type 1** | 68/NF | 1,372,356 | 28,980,311 | 28,980,562 | 30,352,918 | 30,354,679 |
|  | 115/NF | 1,372,356 | 28,980,311 | 28,980,562 | 30,352,918 | 30,354,679 |
|  | 255/NF | 1,372,356 | 28,980,311 | 28,980,562 | 30,352,918 | 30,354,679 |
|  | 428/NF | 1,372,356 | 28,980,311 | 28,980,562 | 30,352,918 | 30,354,679 |
|  | 4672016 | 1,313,885 | 28,992,784 | 28,997,894 | 30,311,779 | 30,311,817 |
|  | 532/NF | 1,352,808 | 29,016,360 | 29,016,594 | 30,369,402 | 30,386,515 |
|  | 629/NF | 1,374,117 | 28,980,311 | 28,980,562 | 30,354,679 | 30,360,494 |
|  | 761/NF | 1,415,063 | 28,980,311 | 28,980,562 | 30,395,625 | 30,398,383 |
| **Type 2** | 85/NF | 1,130,961 | 29,075,205 | 29,075,557 | 30,306,518 | 30,306,552 |
| **Atypical** | 556/NF | 1,122,447 | 29,100,044 | 29,104,296 | 30,226,743 | 30,227,597 |

Genome coordinates shown in table 2 are extracted from ChAS 2.0. The positions were localized on human genome assembly hg38 (GRCh38) which were converted by NCBI Genome Remapping Service and Lift Genome Annotations to genome assembly hg19 (GRCh37).
