# Supplementary material for: Genotype-Phenotype Associations in Patients With Type-1, Type-2, and Atypical NF1 Microdeletions
Source: Front Genet. 2021 Jun 8;12:673025. doi: 10.3389/fgene.2021.673025 (PMC8217751; doi:10.3389/fgene.2021.673025)
Supplement: Supplementary file 3 [file Table_3.docx]

**Supplementary Table 3** Comparison of clinical features observed in patients with *NF1* microdeletions and *NF1* intragenic mutations

| System involvement/manifestations | **Clinical features** | ***NF1* microdeletion patients (n=17)** | ***NF1* non-deleted patients (n=33)** | **p** |
| --- | --- | --- | --- | --- |
| Dysmorphic features | Facial dysmorphism | 9 (53%) | 0 (0%) | **<0,001** |
|  | Hypertelorism | 9 (53%) | 6 (18%) | **0,021** |
|  | Facial asymmetry | 3 (18%) | 2 (6%) | 0,321 |
|  | Coarse face | 9 (53%) | 0 (0%) | **<0,001** |
|  | Broad neck | 1 (6%) | 0 (0%) | 0,340 |
|  | Large hands and feet | 9 (53%) | 0 (0%) | **<0,001** |
| Skin manifestations | Café-au-lait spots | 17 (100%) | 30 (91%) | 0,542 |
|  | Axillary and inguinal freckling | 13 (76%) | 17 (52%) | 0,129 |
|  | Excess soft tissue in hands and feet | 5 (29%) | 0 (0%) | **0,003** |
|  | Subcutaneous neurofibromas | 8 (47%) | 10 (30%) | 0,352 |
|  | Cutaneous neurofibromas | 1 (6%) | 6 (18%) | 0,398 |
|  | Plexiform neurofibromas* | 2 (12%) | 2 (6%) | 0,597 |
| Education and behavior problems | SDiCD | 10 (59%) | 1 (3%) | **<0,001** |
|  | General learning difficulties | 10 (59%) | 5 (15%) | **0,003** |
|  | Speech difficulties | 8 (47%) | 1 (3%) | **<0,001** |
|  | IQ < 70 | 1 (6%) | 0 (0%) | 0,340 |
|  | ADHD | 2 (12%) | 2 (6%) | 0,597 |
| Skeletal manifestations | Skeletal anomalies | 16 (94%) | 11 (33%) | **<0,001** |
|  | Scoliosis | 7 (41%) | 7 (21%) | 0,187 |
|  | Pectus excavatum | 7 (41%) | 3 (9%) | **0,021** |
|  | Bone cysts | 1 (6%) | 0 (0%) | 0,340 |
|  | Hyperflexibility of joints | 1 (6%) | 2 (6%) | 1,000 |
|  | Pes cavus | 0 (0%) | 1 (3%) | 1,000 |
|  | Macrocephaly | 9 (53%) | 3 (9%) | **0,001** |
| Neurological manifestations | Muscular hypotonia | 3 (18%) | 4 (12%) | 0,677 |
|  | Epilepsy | 0 (0%) | 1 (3%) | 1,000 |
|  | MPNST | 2 (12%) | 0 (0%) | 0,111 |
|  | Spinal neurofibromas | 2 (12%) | 1 (3%) | 0,264 |
|  | T2 hyperintensities | 13 (76%) | 13 (39%) | **0,018** |
| Ocular manifestations | Visual disturbance | 3 (18%) | 5 (15%) | 1,000 |
|  | Lisch nodules | 4 (24%) | 7 (21%) | 1,000 |
|  | Strabismus | 2 (12%) | 0 (0%) | 0,111 |
|  | Optic pathway gliomas | 4 (24%) | 4 (12%) | 0,419 |
| Develop. problem | Tall-for-age stature | 7 (41%) | 0 (0%) | **<0,001** |
| Heart problems | Congenital heart defects | 0 (0%) | 0 (0%) | NA |

NA, not applicable; #no straightforward information (only referenced as neurofibroma); * externally observable plexiform neurofibroma, SDiCD, significant delay in cognitive development; MPNST, malignant peripheral nerve sheath tumours; ADHD, attention deficit hyperactivity disorder
