# Supplementary material for: Genotype-Phenotype Associations in Patients With Type-1, Type-2, and Atypical NF1 Microdeletions
Source: Front Genet. 2021 Jun 8;12:673025. doi: 10.3389/fgene.2021.673025 (PMC8217751; doi:10.3389/fgene.2021.673025)
Supplement: Supplementary file 4 [file Table_4.docx]

**Supplementary Table 4** Comparison of clinical features observed in patients with type-1 *NF1* microdeletions and *NF1* intragenic mutations

| System involvement/manifestations | **Clinical features** | ***NF1* microdeletion patients (n=12)** | ***NF1* non-deleted patients (n=33)** | **p** |
| --- | --- | --- | --- | --- |
| Dysmorphic features | Facial dysmorphism | 8 (67%) | 0 (0%) | **<0,001** |
|  | Hypertelorism | 7 (58%) | 6 (18%) | **0,022** |
|  | Facial asymmetry | 3 (25%) | 2 (6%) | 0,109 |
|  | Coarse face | 8 (67%) | 0 (0%) | **<0,001** |
|  | Broad neck | 1 (8%) | 0 (0%) | 0,267 |
|  | Large hands and feet | 8 (67%) | 0 (0%) | **<0,001** |
| Skin manifestations | Café-au-lait spots | 12 (100%) | 30 (91%) | 0,553 |
|  | Axillary and inguinal freckling | 10 (83%) | 17 (52%) | 0,086 |
|  | Excess soft tissue in hands and feet | 4 (33%) | 0 (0%) | **0,003** |
|  | Subcutaneous neurofibromas | 7 (58%) | 10 (30%) | 0,163 |
|  | Cutaneous neurofibromas | 1 (8%) | 6 (18%) | 0,655 |
|  | Plexiform neurofibromas | 2 (17%) | 2 (6%) | 0,286 |
| Education and behavior problems | SDiCD | 9 (75%) | 1 (3%) | **<0,001** |
|  | General learning difficulties | 9 (75%) | 5 (15%) | **<0,001** |
|  | Speech difficulties | 8 (67%) | 1 (3%) | **<0,001** |
|  | IQ < 70 | 1 (8%) | 0 (0%) | 0,267 |
|  | ADHD | 2 (17%) | 2 (6%) | 0,286 |
| Skeletal manifestations | Skeletal anomalies | 11 (92%) | 11 (33%) | **<0,001** |
|  | Scoliosis | 5 (42%) | 7 (21%) | 0,254 |
|  | Pectus excavatum | 5 (42%) | 3 (9%) | **0,022** |
|  | Bone cysts | 1 (8%) | 0 (0%) | 0,267 |
|  | Hyperflexibility of joints | 1 (8%) | 2 (6%) | 1,0 |
|  | Pes cavus | 0 (0%) | 1 (3%) | 1,0 |
|  | Macrocephaly | 7 (58%) | 3 (9%) | **0,01** |
| Neurological manifestations | Muscular hypotonia | 3 (25%) | 4 (12%) | 0,362 |
|  | Epilepsy | 0 (0%) | 1 (3%) | 1,0 |
|  | MPNST | 2 (17%) | 0 (0%) | 0,067 |
|  | Spinal neurofibromas | 2 (17%) | 1 (3%) | 0,169 |
|  | T2 hyperintensities | 10 (83%) | 13 (39%) | **0,017** |
| Ocular manifestations | Visual disturbance | 2 (17%) | 5 (15%) | 1,000 |
|  | Lisch nodules | 3 (25%) | 7 (21%) | 1,000 |
|  | Strabismus | 2 (17%) | 0 (0%) | 0,067 |
|  | Optic pathway gliomas | 2 (17%) | 4 (12%) | 0,650 |
| Develop. problem | Tall-for-age stature | 7 (58%) | 0 (0%) | **<0,001** |
| Heart problems | Congenital heart defects | 0 (0%) | 0 (0%) | NA |

NA, not applicable; #no straightforward information (only referenced as neurofibroma); * externally observable plexiform neurofibroma, SDiCD, significant delay in cognitive development; MPNST, malignant peripheral nerve sheath tumours; ADHD, attention deficit hyperactivity disorder
