# Supplementary material for: Genotype-Phenotype Associations in Patients With Type-1, Type-2, and Atypical NF1 Microdeletions
Source: Front Genet. 2021 Jun 8;12:673025. doi: 10.3389/fgene.2021.673025 (PMC8217751; doi:10.3389/fgene.2021.673025)
Supplement: Supplementary file 5 [file Table_5.docx]

**Supplementary Table 5** Comparison of clinical features observed in patients with type-1 *NF1* microdeletions and *NF1* intragenic mutations in children under 15 years

| System involvement/manifestations | **Clinical features** | ***NF1* microdeletion patients (n=9)** | ***NF1* non-deleted patients (n=20)** | **p** |
| --- | --- | --- | --- | --- |
| Dysmorphic features | Facial dysmorphism | 6 (67 %) | 0 (0 %) | **<0,001** |
|  | Hypertelorism | 6 (67 %) | 6 (30 %) | 0,106 |
|  | Facial asymmetry | 2 (22 %) | 2 (10 %) | 0,568 |
|  | Coarse face | 6 (67 %) | 0 (0 %) | **<0,001** |
|  | Broad neck | 0 (0%) | 0 (0 %) | NA |
|  | Large hands and feet | 7 (78 %) | 0 (0 %) | **<0,001** |
| Skin manifestations | Café-au-lait spots | 9 (100 %) | 20 (100 %) | 1,000 |
|  | Axillary and inguinal freckling | 8 (89 %) | 10 (50 %) | 0,096 |
|  | Excess soft tissue in hands and feet | 3 (33 %) | 0 (0 %) | **0,023** |
|  | Subcutaneous neurofibromas | 5 (56 %) | 3 (15 %) | 0,067 |
|  | Cutaneous neurofibromas | 0 (0 %) | 0 (0 %) | NA |
|  | Plexiform neurofibromas | 0 (0 %) | 1 (5 %) | 1,000 |
| Education and behavior problems | SDiCD | 7 (78 %) | 0 (0 %) | **<0,001** |
|  | General learning difficulties | 6 (67 %) | 3 (15 %) | **0,010** |
|  | Speech difficulties | 7 (78 %) | 0 (0 %) | **<0,001** |
|  | IQ < 70 | 0 (0 %) | 0 (0 %) | NA |
|  | ADHD | 1 (11 %) | 1 (5 %) | 0,532 |
| Skeletal manifestations | Skeletal anomalies | 8 (89 %) | 7 (35 %) | **0,014** |
|  | Scoliosis | 0 (0 %) | 2 (10 %) | 1,000 |
|  | Pectus excavatum | 3 (33 %) | 2 (10 %) | 0,287 |
|  | Bone cysts | 0 (0 %) | 0 (0 %) | NA |
|  | Hyperflexibility of joints | 1 (11 %) | 2 (10 %) | 1,000 |
|  | Pes cavus | 0 (0 %) | 1 (5 %) | 1,000 |
|  | Macrocephaly | 6 (67 %) | 3 (15 %) | 0,010 |
| Neurological manifestations | Muscular hypotonia | 2 (22 %) | 4 (20 %) | 1,000 |
|  | Epilepsy | 0 (0%) | 0 (0 %) | NA |
|  | MPNST | 0 (0 %) | 0 (0 %) | NA |
|  | Spinal neurofibromas | 1 (11 %) | 0 (0 %) | 0,310 |
|  | T2 hyperintensities | 6 (67 %) | 11 (55 %) | 0,694 |
| Ocular manifestations | Visual disturbance | 0 (0 %) | 2 (10 %) | 1,000 |
|  | Lisch nodules | 0 (0 %) | 3 (15 %) | 0,532 |
|  | Strabismus | 2 (22 %) | 0 (0 %) | 0,089 |
|  | Optic pathway gliomas | 1 (11 %) | 3 (15 %) | 1,000 |
| Develop. problem | Tall-for-age stature | 5 (56 %) | 0 (0 %) | **0,001** |
| Heart problems | Congenital heart defects | 0 (0 %) | 0 (0 %) | NA |

NA, not applicable; #no straightforward information (only referenced as neurofibroma); * externally observable plexiform neurofibroma, SDiCD, significant delay in cognitive development; MPNST, malignant peripheral nerve sheath tumours; ADHD, attention deficit hyperactivity disorder
